# Cefiderocol Treatment for Patients with Multidrug- and Carbapenem-Resistant Pseudomonas aeruginosa Infections in the Compassionate Use Program

**DOI:** 10.1128/aac.00194-23

**Published:** 2023-06-22

**Authors:** Michael J. Satlin, Patricia J. Simner, Christine M. Slover, Yoshinori Yamano, Tsutae D. Nagata, Simon Portsmouth

**Affiliations:** a Transplant-Oncology Infectious Diseases Program, Division of Infectious Diseases, Department of Medicine, Weill Cornell Medicine, New York, New York, USA; b Department of Pathology, Johns Hopkins University School of Medicine, Baltimore, Maryland, USA; c Shionogi Inc., Florham Park, New Jersey, USA; d Shionogi & Co., Ltd., Osaka, Japan

**Keywords:** cefiderocol, clinical response, compassionate use, carbapenem-resistant *Pseudomonas aeruginosa*, susceptibility breakpoint

## Abstract

Cefiderocol is an option for infections caused by multidrug-resistant Pseudomonas aeruginosa, but its *in vitro* activity against these isolates and its clinical effectiveness for isolates with MICs of >1 μg/mL is unclear. We investigated the *in vitro* activity of cefiderocol against P. aeruginosa isolates collected from patients treated with cefiderocol through the compassionate use program and assessed physician-reported clinical response and 28-day all-cause mortality by cefiderocol MIC values. P. aeruginosa isolates underwent susceptibility testing to cefiderocol and comparator agents by using reference broth microdilution. U.S. Food and Drug Administration (FDA; susceptible, ≤1 μg/mL) and Clinical and Laboratory Standards Institute (CLSI; susceptible, ≤4 μg/mL) cefiderocol breakpoints were applied. Additionally, molecular characterization of β-lactamase genes was performed. Clinical response and vital status were reported by treating physicians. Forty-six patients with P. aeruginosa infections were evaluated. Twenty-nine (63%) and 42 (91%) isolates were susceptible to cefiderocol using FDA and CLSI breakpoints, respectively. Thirty-seven (80%) and 32 (70%) isolates were not susceptible to ceftolozane-tazobactam and ceftazidime-avibactam, respectively. The clinical response rate was 69% (20/29) with a cefiderocol MIC of ≤1 μg/mL, 69% (9/13) with a cefiderocol MIC of 2 to 4 μg/mL, and 100% (4/4) with an MIC of ≥8 μg/mL, while day 28 all-cause mortality rates were 23% (6/26; MIC ≤ 1 μg/mL), 33% (4/12; MIC, 2 to 4 μg/mL), and 0% (0/4; MIC ≥8 μg/mL), respectively. Cefiderocol was active *in vitro* against most P. aeruginosa isolated from patients with limited or no alternative therapies. Patients with cefiderocol MICs of 2 to 4 μg/mL did not have significantly worse outcomes than those with MICs of ≤1 μg/mL.

## INTRODUCTION

Multidrug-resistant (MDR) and carbapenem-resistant (CR) Pseudomonas aeruginosa infections are associated with poor outcomes and increased risk of mortality compared with infections caused by susceptible bacteria (1–3). The prevalence of CR P. aeruginosa can reach 40 to 50% in certain regions (4, 5). MDR P. aeruginosa was reported to be associated with a mortality rate of 25 to >40% in patients with ventilator-associated pneumonia and bloodstream infection (3, 6, 7). MDR and CR P. aeruginosa infections have limited treatment options, with newer β-lactam–β-lactamase inhibitors (BL-BLIs, including ceftolozane-tazobactam, ceftazidime-avibactam, and imipenem-relebactam) and cefiderocol being recommended by the Infectious Diseases Society of America for complicated urinary tract infections and infections outside the urinary tract (8). However, resistance to new BL-BLIs has become increasingly common among MDR and CR P. aeruginosa, particularly with the global emergence of carbapenemases that often confer resistance to these agents (9, 10). Recent studies indicate that 11 to 46% of MDR and CR P. aeruginosa isolates are not susceptible to these new BL-BLIs (9–11). Cefiderocol, however, retains *in vitro* activity against most P. aeruginosa isolates that are resistant to new BL-BLIs, including carbapenemase-producing isolates (10–12).

Cefiderocol has been approved by the U.S. Food and Drug Administration (FDA) for the treatment of complicated urinary tract infection and nosocomial pneumonia, including ventilator-associated pneumonia, caused by susceptible aerobic Gram-negative bacteria (13). In Europe, cefiderocol has been approved by the European Medicines Agency for the treatment of infections caused by susceptible Gram-negative bacteria with limited treatment options (14). The efficacy and safety of cefiderocol have been demonstrated in phase 2 and phase 3 clinical trials in patients with serious Gram-negative infections (15–17), but patients in these studies typically had other potential treatment options that allowed for randomization and trial enrollment. Thus, these trials provide limited information on the *in vitro* and clinical effectiveness of cefiderocol for patients infected with P. aeruginosa without alternative treatment options.

In addition to limited clinical data for MDR and CR P. aeruginosa infections, the optimal cefiderocol susceptibility breakpoint for P. aeruginosa is uncertain. Pharmacokinetic/pharmacodynamic (PK/PD) modeling suggests that the pharmacodynamic target for P. aeruginosa that correlates with *in vivo* killing in animal models is likely to be achieved when the cefiderocol MIC value is ≤4 μg/mL (18, 19). However, few patients in clinical trials of cefiderocol were infected with P. aeruginosa isolates with MIC values >1 μg/mL (15–17). This uncertainty is reflected by differences in cefiderocol breakpoints with P. aeruginosa: the FDA susceptible breakpoint is ≤1 μg/mL, the Clinical and Laboratory Standards Institute (CLSI) susceptible breakpoint is ≤4 μg/mL, and the European Committee on Antimicrobial Susceptibility Testing (EUCAST) susceptible breakpoint is ≤2 μg/mL (see Table S1 in the supplemental material) (20–22).

Cefiderocol was administered via the compassionate use program to 251 patients between April 2016 and November 2020 prior to marketing authorization in the United States and Europe (23, 24). This program allowed patients with life-threatening infections to be treated with cefiderocol if they had no alternative options because of extensive antimicrobial resistance or established toxicity of existing antibiotics. The aims of the current analysis were to (i) evaluate the *in vitro* activity of cefiderocol and comparator agents against MDR and CR P. aeruginosa isolates from the compassionate use program, (ii) characterize the β-lactamase gene content of these isolates, and (iii) assess clinical outcomes with cefiderocol therapy for infections with different cefiderocol MIC values.

(Data included in the manuscript were presented in part as a poster at IDWeek 2021 virtual congress [25]).

## RESULTS

### Compassionate use of cefiderocol.

**FIG 1:**
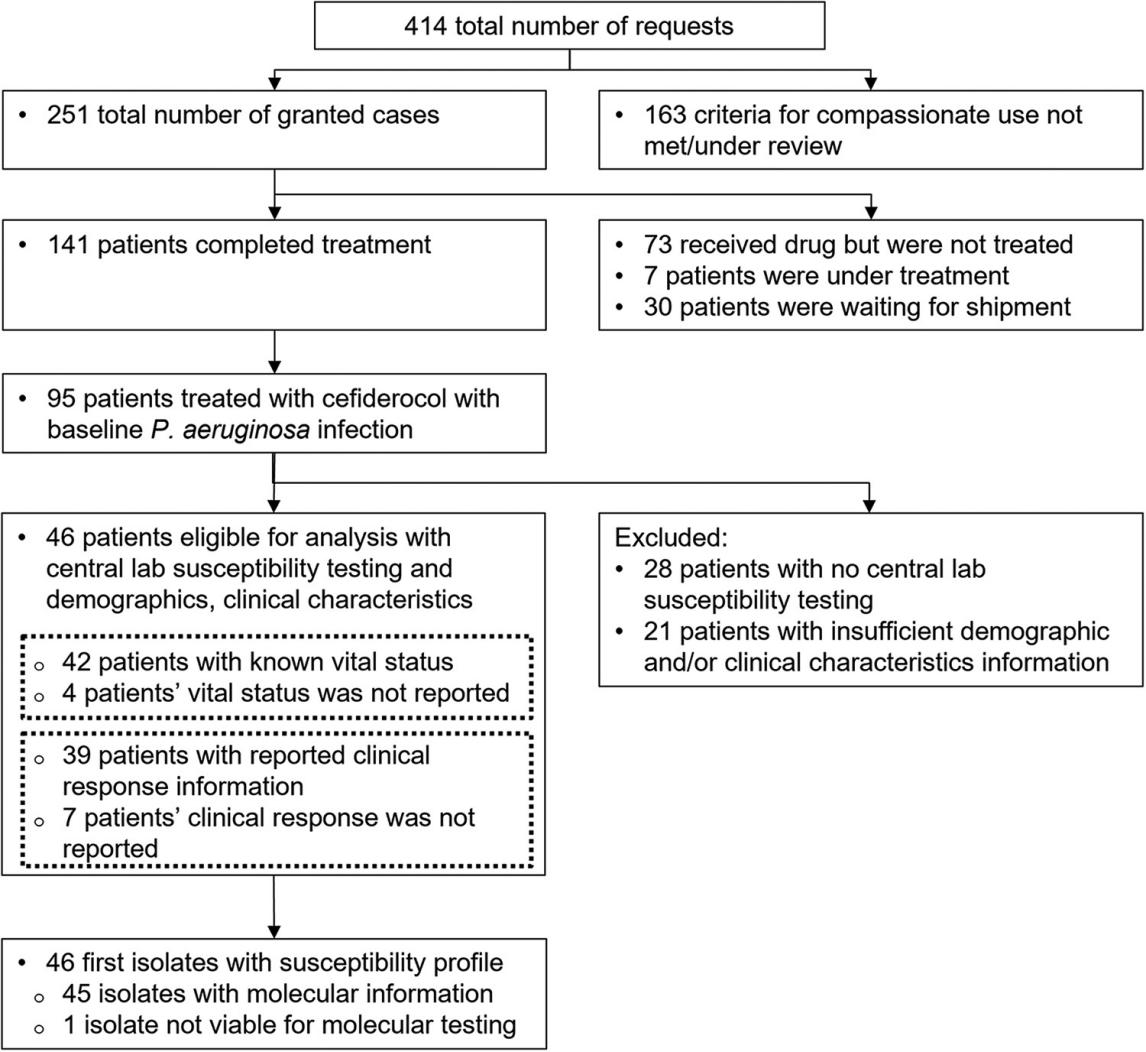
Patient flow diagram and study eligibility of cases with Pseudomonas aeruginosa infections under compassionate use of cefiderocol.

Between April 2016 and November 2020, 414 requests were received and 251 were granted to receive treatment with cefiderocol under the compassionate use program in the United States, Canada, and Europe (Fig. 1). Of these 251 cases, 95 patients with P. aeruginosa infection were treated with cefiderocol. Of these 95 patients, 28 were excluded because susceptibility testing was not performed by the central laboratory, and 21 were excluded because of insufficient clinical information, leaving a final study cohort of 46 patients (Fig. 1).

### Clinical information for the study cohort.

Forty-two patients (91%) were treated in the United States and Canada, and four (9%) were treated in Europe. The baseline characteristics of patients are shown in Table 1. The median age was 51.5 (range, 15 to 83) years, and 31 patients (67%) were male. Infections at more than one anatomical site were present in 20 patients (43%). The three most common types of infection were respiratory tract infection (*n *= 23; 50%), bloodstream infection (*n *= 15; 33%), and wound infection (*n *= 10; 22%). The most common comorbid conditions were pulmonary and respiratory diseases (*n *= 22; 48%), acute or chronic kidney disease (*n *= 20; 43%), and cardiovascular disease (*n *= 12; 26%). Ten patients (22%) had cystic fibrosis, 10 (22%) had a transplant (e.g., lung, kidney, liver, heart) and were receiving immunosuppressive therapies, and 4 (9%) had cancer. Twenty (43%) patients had additional coinfecting pathogens.

**TABLE 1:** Baseline clinical characteristics of 46 patients treated with cefiderocol in the compassionate use program for a multidrug- or carbapenem-resistant Pseudomonas aeruginosa infection

Characteristic	Data for all patients (*n* = 46)
Age (median [range]^*g*^ [yrs])	51.5 (15–83)
Sex (no. [%])	
Male	31 (67)
Female	15 (33)
Infection type (no. [%])^*a*^	
Respiratory tract infection	23 (50)
Bloodstream infection	15 (33)
Wound infection	10 (22)
Intra-abdominal infection	9 (20)
Bone or joint infection	8 (17)
Urinary tract infection	7 (15)
Sepsis	5 (11)
Single anatomical site of infection	26 (57)
Multiple sites of infection	20 (43)
Comorbidities, medical history (no. [%])^*b*^	
Pulmonary/respiratory	22 (48)
Renal disease	20 (43)
Cardiovascular disease	12 (26)
Cystic fibrosis	10 (22)
Transplant	10 (22)
Diabetes	7 (15)
Cancer	4 (9)
Obesity	2 (4)
Baseline coinfecting pathogen (no. [%])	20 (43)
Achromobacter xylosoxidans	3 (7)
Klebsiella pneumoniae	3 (7)
Acinetobacter baumannii	2 (4)
Other Gram-negative pathogens^*c*^	5 (11)
Mycobacterium spp. (nontuberculous)^*d*^	3 (7)
VRE^*e*^	3 (7)
Staphylococcus spp.	2 (4)
Bacteroides fragilis	1 (2)
*Candida* spp.	1 (2)
Concomitant antibiotics (no. [%])	
Gram-negative antibiotics^*f*^	38 (83)
Gram-positive antibiotics^*f*^	19 (41)
Antiviral agents	1 (2)
Antifungal agents	9 (20)
Antianaerobe agents	6 (13)
Bacteriophage therapy (no. [%])	1 (2)
Cefiderocol treatment duration (median [range]^*g*^[days])	22.5 (1–132)

a Patients could have had ≥1 concurrent infection prior to cefiderocol treatment.

b Patients could have had ≥1 comorbid conditions.

c Includes Stenotrophomonas maltophilia (*n *= 1), Burkholderia cepacia complex (*n *= 1), Escherichia coli (*n *= 1), Citrobacter koseri (*n *= 1), and Morganella morganii (*n *= 1).

d Includes M. abscessus (*n *= 2) and species unspecified (*n *= 1).

e VRE, vancomycin-resistant *Enterococcus*. Includes Enterococcus faecium (*n *= 2) and species unspecified (*n *= 1).

f Further details are included in Table S2 in the supplemental material.

g Range indicates minimum and maximum.

Cefiderocol treatment was administered for a median of 22.5 days (range, 1 to 132 days). The most frequently administered concomitant antibiotics were polymyxins (*n *= 22; 48%), aminoglycosides (*n *= 11; 24%), carbapenems (*n *= 10; 22%), and newer BL-BLI agents (*n *= 9; 20%) and eravacycline (*n *= 3; 7%) (Table S2). Nearly all patients received antibiotics, including ceftolozane-tazobactam and ceftazidime-avibactam, prior to the use of cefiderocol and received concomitant antibiotics during cefiderocol therapy.

### Antimicrobial susceptibility profiles of P. aeruginosa isolates.

The susceptibility profiles of the 46 baseline P. aeruginosa isolates are shown in Table 2. The majority of isolates were resistant to meropenem (85%), imipenem (81%), ciprofloxacin (89%), cefepime (67%), and aztreonam (60%), and one (2%) was resistant to colistin (Table 2). Forty-four of 46 isolates (96%) were characterized as MDR P. aeruginosa. Three isolates were not designated CR because they were susceptible to meropenem and imipenem, but the patients had another MDR and CR Gram-negative pathogen at baseline (MDR Acinetobacter baumannii complex [cefiderocol MIC, 2 μg/mL], extensively drug-resistant Achromobacter xylosoxidans [cefiderocol MIC, 0.06 μg/mL] plus Mycobacterium abscessus, or MDR Klebsiella pneumoniae [cefiderocol MIC, unknown]), which made them eligible for compassionate use of cefiderocol. Thirty-seven isolates (80%) were not susceptible to ceftolozane-tazobactam, and 32 (70%) were resistant to ceftazidime-avibactam (Table 2).

**TABLE 2:** Antimicrobial susceptibility profiles of 46 baseline multidrug-resistant or carbapenem-resistant Pseudomonas aeruginosa isolates from 46 patients receiving cefiderocol in the compassionate use program by using CLSI breakpoints^*a*^

Antimicrobial agent	MIC^*b*^_90_ (μg/mL)	MIC range (μg/mL)	Interpretation (no. [%])		
			S	I	R
Cefiderocol^*c*^^,^^*d*^ (*n *= 46)	4	≤0.03 to 16	42 (91)	3 (7)	1 (2)
Amikacin (*n* = 43)	>64	≤4 to >64	22 (51)	7 (16)	14 (33)
Aztreonam (*n* = 43)	>32	2 to >32	5 (12)	12 (28)	26 (60)
Cefepime (*n* = 46)	>16	2 to >64	4 (9)	11 (24)	31 (67)
Ceftazidime-avibactam (*n* = 46)	>64	1 to >64	14 (30)	NA	32 (70)
Ceftolozane-tazobactam (*n* = 46)	>64	0.5 to >64	9 (20)	2 (4)	35 (76)
Ciprofloxacin (*n* = 46)	>4	≤0.25 to >8	3 (7)	2 (4)	41 (89)
Colistin (*n* = 46)	2	≤0.5 to >8	NA	45 (98)	1 (2)
Imipenem^*e*^ (*n* = 43)	>64	1 to >64	7 (16)	1 (2)	35 (81)
Meropenem^*e*^ (*n* = 46)	>64	0.25 to >64	4 (9)	3 (7)	39 (85)

a CLSI, Clinical and Laboratory Standards Institute; FDA, U.S. Food and Drug Administration; I, intermediate; R, resistant; S, susceptible; NA, not applicable.

b MIC values were obtained by broth microdilution method at a central laboratory according to CLSI.

c Four patients had P. aeruginosa isolates with cefiderocol MIC values of >4 μg/mL. These four patients were eligible for compassionate use based on the following reasons: one patient with a cefiderocol MIC of 16 μg/mL had CR Acinetobacter baumannii coinfection with no alternative treatment option, one patient with a cefiderocol MIC of 8 μg/mL had Achromobacter xylosoxidans coinfection with no alternative treatment option, and two patients with cefiderocol MICs of 8 μg/mL had a locally tested cefiderocol-susceptible P. aeruginosa isolate before treatment initiation, and later, the central laboratory confirmed cefiderocol resistance in the current analysis.

d Based on FDA breakpoints (S, ≤1 μg/mL; I, 2 μg/mL; R, ≥4 μg/mL) as follows: susceptible, 63%, *n *= 29; intermediate, 20%, *n *= 9; and resistant, 17%, *n *= 8.

e Three isolates were susceptible to meropenem and imipenem, but the patient had another carbapenem-resistant Gram-negative pathogen at baseline (A. baumannii, *n *= 1; A. xylosoxidans: *n *= 1; and Klebsiella pneumoniae, *n *= 1).

**FIG 2:**
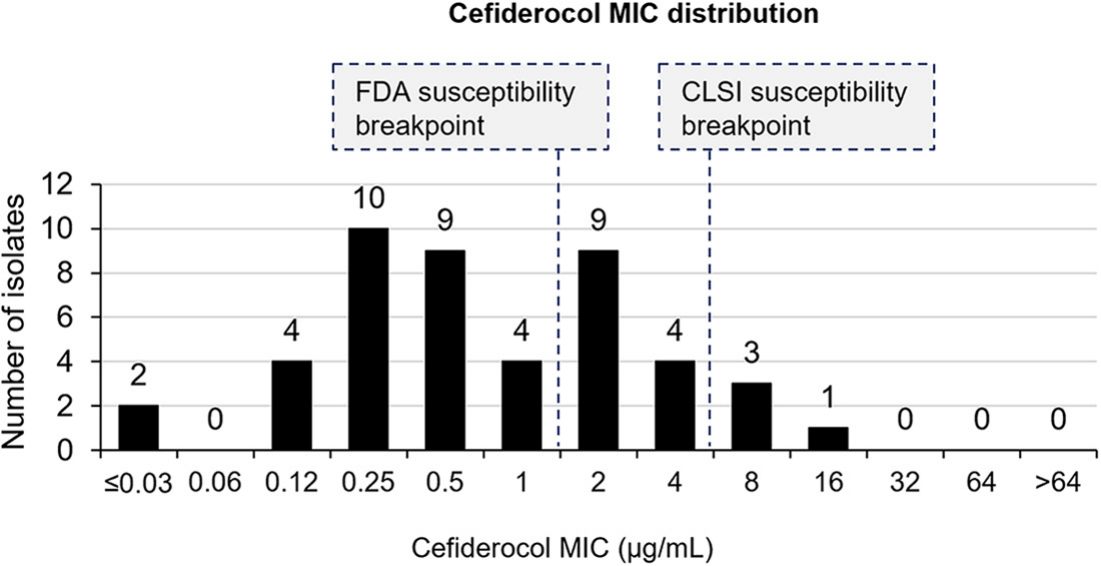
Cefiderocol MIC distribution of 46 multidrug- or carbapenem-resistant Pseudomonas aeruginosa isolates treated with cefiderocol under the compassionate use program. CLSI, Clinical and Laboratory Standards Institute; FDA, U.S. Food and Drug Administration.

Cefiderocol MIC_50_ and MIC_90_ values were 0.5 μg/mL and 4 μg/mL, respectively. MICs ranged between ≤0.03 μg/mL and 16 μg/mL, and four isolates had cefiderocol MICs ≥8 μg/mL (Fig. 2). Based on FDA breakpoints, 63% of isolates (*n *= 29) were susceptible, 20% (*n *= 9) were intermediate, and 17% (*n *= 8) were resistant to cefiderocol. Based on CLSI breakpoints, 91% (*n *= 42) were susceptible, 7% (*n *= 3) were intermediate, and 2% (*n *= 1) were resistant to cefiderocol.

All nine ceftolozane-tazobactam-susceptible P. aeruginosa isolates had cefiderocol MIC values of <0.5 μg/mL and thus were susceptible to cefiderocol based on CLSI and FDA breakpoints (Fig. S1). Of the 37 isolates not susceptible to ceftolozane-tazobactam, 20 (54%) had cefiderocol MIC values of ≤1 μg/mL (FDA susceptible breakpoint), and 33 (89%) had cefiderocol MIC values of ≤4 μg/mL (CLSI susceptible breakpoint). Two (14%) of the 14 ceftazidime-avibactam-susceptible isolates were not susceptible to cefiderocol according to CLSI and FDA breakpoints (MIC = 8 μg/mL) (Fig. S2). Of the 32 ceftazidime-avibactam-resistant isolates, 18 (56%) had cefiderocol MIC values of ≤1 μg/mL (FDA susceptible breakpoint), and 30 (94%) had cefiderocol MIC values of ≤4 μg/mL (CLSI susceptible breakpoint).

A total of 13 P. aeruginosa isolates had cefiderocol MIC values of 2 and 4 μg/mL (i.e., not susceptible by the FDA breakpoint but susceptible by the CLSI breakpoint). All were resistant to ceftolozane-tazobactam (MICs ≥ 64 μg/mL) and meropenem (MICs, 4 to ≥64 μg/mL), and all but one were resistant to ceftazidime-avibactam (MICs ≥ 32 μg/mL; the susceptible isolate had a ceftazidime-avibactam MIC value of 2 μg/mL).

### Molecular characterization of P. aeruginosa isolates.

Molecular characterization was performed on 45 of 46 isolates. One isolate was not viable for further characterization. Molecular analysis provided information on the presence of acquired β-lactamase genes and chromosomally encoded Pseudomonas-derived cephalosporinase (PDC) variants, as detailed below (Table S2).

**(i) Beta-lactamases.** Nine (20%) isolates harbored the following class A β-lactamase genes: *bla*_GES_ (*n *= 5, including *bla*_GES-1_ [*n *= 3], *bla*_GES-7_ [*n *= 1], and *bla*_GES-40_ [*n *= 1]), *bla*_VEB-9_ (*n *= 3), and *bla*_PER-1_ (*n *= 1). No class A *bla*_CTX-M_, *bla*_SHV_, or *bla*_TEM_ extended-spectrum β-lactamase (ESBL) or *bla*_KPC_ genes were detected. Nine (20%) isolates harbored the following class B metallo-β-lactamase (MBL) genes: *bla*_NDM-1_ (*n *= 4), *bla*_VIM-2_ (*n *= 3), *bla*_IMP-15_ and *bla*_VIM-2_ (*n *= 1), and *bla*_IMP-18_ and *bla*_VIM-2_ (*n *= 1). No acquired AmpC β-lactamase genes were identified, but class C PDC genes were detected in all isolates (Table S2).

Cefiderocol MIC values were ≤4 μg/mL in all nine MBL-producing isolates. All nine isolates had meropenem and imipenem MIC values of ≥64 μg/mL and were resistant to ceftolozane-tazobactam, and eight of nine isolates were resistant to ceftazidime-avibactam. Among 13 isolates that were not susceptible to cefiderocol by FDA criteria but susceptible by CLSI criteria (MICs, 2 to 4 μg/mL), 5 isolates carried an acquired β-lactamase gene (bla_NDM-1_ [*n *= 3], *bla*_VEB-9_ and *bla*_NDM-1_ [*n *= 1], and *bla*_GES-7_ [*n *= 1]).

**(ii) PDC variants and antimicrobial susceptibility.** Twenty-four different PDC variants were detected in the 45 isolates, of which 8 were new variants. *bla*_PDC-3_ was the most frequent gene (*n *= 8), followed by *bla*_PDC-19A_ (*n *= 5) (Table S2).

The number of amino acid variations among the PDC variants ranged between one and six compared with wild-type PDC-1 (Table S2). Seven isolates had a group 1 alteration (E247K, G242S, V239A) of PDC, one had a group 2 alteration (M318L), and three had a group 3 alteration (F147L, P180L); however, one isolate had both group 1 and group 3 alterations (Table S3). In one isolate, PDC-new variant 2 harbored the R324del mutation near the R2 loop region.

Of the 10 isolates with group 1 and/or group 3 PDC changes, 3 had cefiderocol MIC values of 8 to 16 μg/mL, and 4 had cefiderocol MIC values of 2 to 4 μg/mL. All of these isolates were resistant to cefepime, aztreonam, and ceftolozane-tazobactam, nine were resistant to meropenem, eight were resistant to ceftazidime-avibactam, and six were resistant to imipenem (Table S3). None of the 11 MBL and GES carbapenemase-producing Pseudomonas isolates had group 1 to 3 changes in their PDC enzymes. The susceptibilities of isolates without a carbapenemase, ESBL, or acquired AmpC β-lactamase to cefiderocol, ceftolozane-tazobactam, and ceftazidime-avibactam are stratified by PDC type in Table S4.

### Clinical outcomes by MIC and by molecular information.

Clinical response rates by cefiderocol MIC values are shown in Table 3. Thirty-three patients (72%) had a clinical response to cefiderocol treatment (with improvement or stabilization or cure and discharge), six (13%) did not respond, and seven (15%) did not have clinical response reported. There was no correlation between cefiderocol MIC value and clinical response.

**TABLE 3:** Physician-reported clinical response for 46 patients treated with cefiderocol via the compassionate use program for an infection due to multidrug- or carbapenem-resistant Pseudomonas aeruginosa, stratified by cefiderocol MIC value

MIC (μg/mL)	No. of patients	Physician-reported response (no. [row %])		
		Response	No response/withdrawn from therapy	No report/unknown
≤0.03	2	1 (50)	1 (50)	0
0.06	0	0	0	0
0.12	4	4 (100)	0	0
0.25	10	6 (60)	2 (20)	2 (20)
0.5	9	6 (67)	1 (11)	2 (22)
1	4	3 (75)	0	1 (25)
2	9	5 (56)	2 (22)	2 (22)
4	4	4 (100)	0	0
8	3	3 (100)	0	0
16	1	1 (100)	0	0
≥32	0	0	0	0
Total patients^*a*^	46	33 (72)	6 (13)	7 (15)

a If multiple baseline P. aeruginosa isolates with different cefiderocol MIC values were identified, the isolate with the highest cefiderocol MIC value was used.

**FIG 3:**
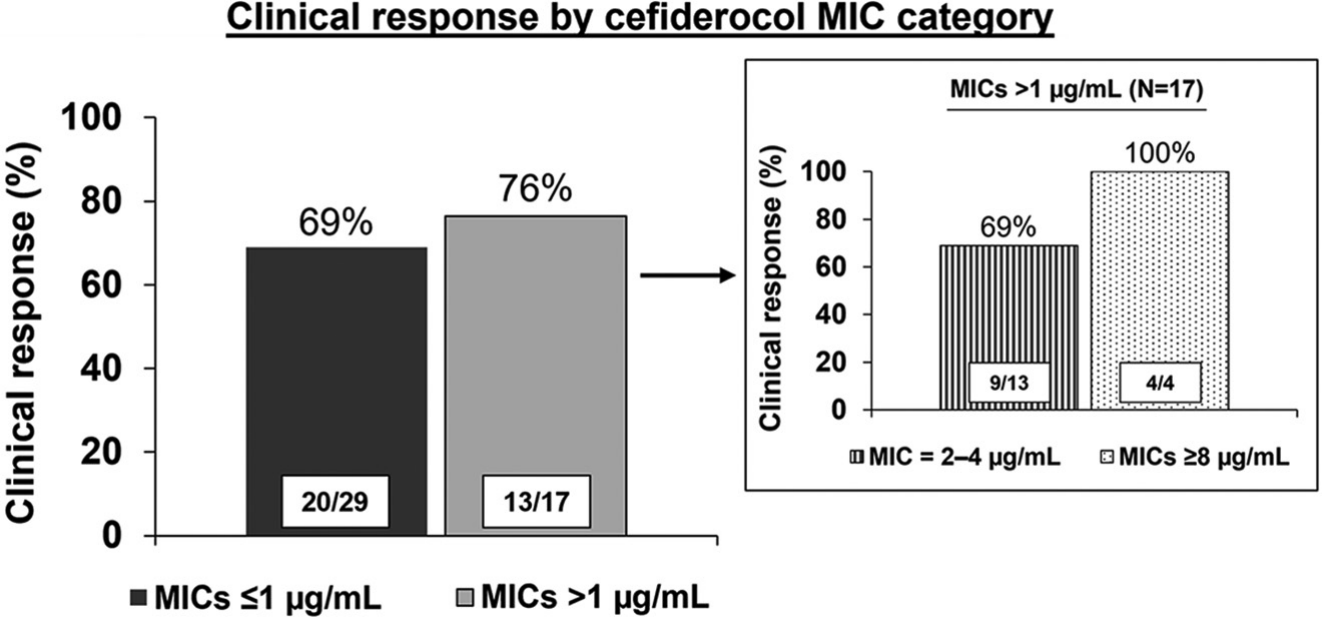
Physician-reported clinical response to cefiderocol therapy in the compassionate use program by cefiderocol MIC category. Ninety-five percent confidence intervals determined by the Clopper-Pearson method as follows: MIC of ≤1 μg/mL, 49.17 to 84.72%; MIC of >1 μg/mL, 50.10 to 93.19%; MIC of 2 to 4 μg/mL, 38.57 to 90.91%; and MIC of ≥8 μg/mL, 39.76 to 100%.

Among 29 patients with cefiderocol MIC values of ≤1 μg/mL (FDA susceptible breakpoint), 20 (69%) responded, 4 (14%) did not respond, and 5 (17%) did not have clinical response reported (Fig. 3). Among 13 patients with cefiderocol MIC values of 2 and 4 μg/mL (not susceptible by FDA breakpoints but susceptible by CLSI breakpoints), 9 (69%) had a clinical response, 2 (15%) did not respond, and 2 (15%) did not have a clinical response reported. All four patients with cefiderocol MIC values of 8 or 16 μg/mL (not susceptible by both FDA and CLSI breakpoints) responded to cefiderocol treatment. Clinical response was reported for six of nine patients (67%) with an MBL-positive P. aeruginosa and was unknown for the remaining three (33%) of these patients.

### Mortality at day 28 and adverse events.

Of the 42 patients with known vital status at day 28, 10 (24%) had died. None of the deaths were reported by the treating physician to be related to cefiderocol. Of these patients with known vital status, 6 of the 26 patients (23.1%) with cefiderocol MIC values of ≤1 μg/mL and 4 of the 12 patients (33.3%) with cefiderocol MIC values of 2 to 4 μg/mL died by day 28. None of the patients (0%) with cefiderocol MIC values of 8 to 16 μg/mL died.

Adverse events were reported for 19 patients (41%). Three of these patients had adverse events related or possibly related to cefiderocol: one patient developed elevated liver enzymes with confounding factors (liver transplant, hepatic abscesses postsurgery) that resolved after discontinuation of cefiderocol, one patient developed Clostridioides difficile infection (resolved following intervention with oral vancomycin, fecal transplantation of microbiota) and thrombocytopenia (resolved following completion of cefiderocol treatment), and one patient had a possibly related acute interstitial nephritis with confounding factors (chronic kidney diseases and obstructive uropathy), which was resolved after discontinuation of cefiderocol.

## DISCUSSION

This analysis of patients with MDR and CR P. aeruginosa infections with limited treatment options who participated in the compassionate use program showed that an identical proportion (69%) of patients responded to cefiderocol treatment when cefiderocol MIC values were ≤1 μg/mL (the FDA susceptible breakpoint) compared with 2 to 4 μg/mL (susceptible by CLSI breakpoints but not by FDA) (Fig. 3). Although patients with cefiderocol MIC values of 2 to 4 μg/mL had numerically higher mortality at day 28 (33%) than patients with cefiderocol MIC values of ≤1 μg/mL (23%), this difference was not statistically significant. Furthermore, all four patients with cefiderocol MICs of 8 to 16 μg/mL survived, suggesting lack of an association between MIC values and mortality. The differences in FDA and CLSI susceptibility breakpoints led to major disparities in the proportion of these highly resistant organisms that were susceptible to cefiderocol: 63% were susceptible using the FDA breakpoints, and 91% were susceptible using CLSI breakpoints.

Although the clinical outcomes data collected during the compassionate use program were limited, it is noteworthy that these outcomes were similar between cefiderocol-treated patients infected with isolates with MIC values of 2 to 4 μg/mL and those infected with isolates with MIC values of ≤1 μg/mL. These clinical data complement data demonstrating *in vivo* efficacy in animal models of infection. In murine thigh and lung infection models of P. aeruginosa and other glucose nonfermenters, humanized dosing of cefiderocol resulted in >1-log reduction in bacterial burden, with cefiderocol MIC values of up to 4 μg/mL (26–28). Surprisingly, all four cefiderocol-treated patients who were infected with organisms with MIC values of 8 and 16 μg/mL had clinical responses and 28-day survival. We do not know whether this surprising finding is related to chance, concomitant medications, decreased virulence or fitness of the infecting organisms, sufficient exposures of cefiderocol at the sites of infection, or host factors.

In the randomized phase 3 clinical studies, all-cause mortality rates in P. aeruginosa infections were similar to those observed in this cohort. In the CREDIBLE-CR study in the cefiderocol arm, 4 (24%) of 17 patients with MDR and CR P. aeruginosa infections died within 28 days, and 6 (35%) of these patients died by end of study (17). In the cefiderocol arm of the APEKS-NP study, 8% of patients with carbapenem-susceptible P. aeruginosa pneumonia died by day 28 (16). The clinical cure rates by test of cure were 58% and 67%, respectively, in these two trials (16, 17). Case reports have demonstrated that patients with infections caused by cefiderocol-susceptible (MICs, 2 to 4 μg/mL; applying CLSI breakpoint) P. aeruginosa and no alternative treatment options responded to cefiderocol treatment with clinical cure; however, clinical failure or death was reported for five patients with cefiderocol-resistant (MICs, 8 to 32 μg/mL) P. aeruginosa infections (29).

Ceftolozane-tazobactam and ceftazidime-avibactam are established first-line therapies for MDR and CR P. aeruginosa (8). The current study included patients whose infections were largely resistant to these new agents and for whom additional therapies are urgently needed. We have found that cefiderocol was active *in vitro* against most isolates that were resistant to ceftolozane-tazobactam and ceftazidime-avibactam, particularly when applying the CLSI cefiderocol breakpoints. This collection of P. aeruginosa isolates included isolates that harbored MBL enzymes (e.g., NDM, VIM, IMP) and other carbapenemases (e.g., GES-7, GES-40). Our findings complement those of two studies in Europe that showed that 100% of MBL-producing P. aeruginosa isolates were susceptible to cefiderocol (30, 31). Therefore, cefiderocol represents an important therapeutic option for infections caused by these organisms.

The patient population analyzed was a heterogeneous group with serious underlying conditions, who had been heavily pretreated with several antibiotics within 90 days prior to compassionate use of cefiderocol. Approximately half of the patients had other pathogens and multiple infections simultaneously. Cefiderocol was requested as a salvage antibiotic either due to lack of activity or established toxicity of other available antibiotics. The median duration of cefiderocol therapy was >3 weeks. The longest treatment duration among the analyzed patients was 132 days (adverse events reported include C. difficile diarrhea, which resolved with fecal microbiota transplantation, and thrombocytopenia, which resolved after treatment completion). Despite this, cefiderocol treatment was well tolerated. Adverse events were rarely attributed to cefiderocol and resolved after either discontinuation or an intervention.

The association between mutations in the chromosomal PDC enzyme and susceptibility to newer beta-lactam antibiotics is of high interest. In this study, isolates without an acquired carbapenemase had PDC variants that led to resistance to newer BL-BLI antibiotics; similar observations have previously been reported (32, 33). Overall, PDC variants with group 1, 2, or 3 mutations, originally described by Berrazeg et al. (34), seemed to show high resistance to ceftolozane-tazobactam and ceftazidime-avibactam, as previously reported, but the mutations had no direct association with cefiderocol susceptibility. It was also observed that many of the isolates with PDC variants with group 0 substitutions also showed high resistance to ceftolozane-tazobactam and ceftazidime-avibactam, particularly when the number of amino acid substitutions was greater. A previous study has shown that certain mutations in PDC-1 and PDC-3 could lead to 8- to 32-fold increases in cefiderocol MIC, along with resistance development to ceftolozane-tazobactam and ceftazidime-avibactam (33). However, mutations in PDC-1 or PDC-3 or in their regulator proteins may not result in cefiderocol resistance, and increased MIC values remain below the CLSI susceptibility breakpoint (33).

Cefiderocol resistance may also emerge due to mutations occurring in iron transport-related proteins (i.e., transposon integration into the *piuA* gene, deletion of *piuD* or *pirA*, premature stop codon affecting the *pirR* gene, or overexpression of the *pvdS*, *fecI*, and *fecA* genes) (33, 35). It has been postulated that resistance to cefiderocol may require multiple simultaneous mechanisms to be present in Gram-negative bacteria (33, 36). Thus, mutations in iron transport-related genes cannot be ruled out for cefiderocol-resistant isolates in the current work.

One limitation of this investigation was that imipenem-relebactam susceptibility was not investigated by the central laboratory for these P. aeruginosa isolates; thus, some of these isolates might have been susceptible to imipenem-relebactam, offering a potential alternative treatment option as previously shown (33). Additionally, the compassionate use program did not have a precise definition of clinical response for the different infection types. Information was provided at the discretion of the treating physicians, although a report of death as a serious adverse event was mandatory. Another limitation was that whole-genome sequencing was not conducted; therefore, more details on the molecular profile of the baseline (prior to treatment) P. aeruginosa isolates could not be collected (e.g., mutations in iron transport genes). Thorough investigation of the association between susceptibility profile at baseline and prior antibiotic treatment was not possible because of the combination of antibiotics with coverage against Gram-negative bacteria and due to lack of precise information on the duration of prior treatment or a specified time frame to assess clinical response.

### Conclusion.

The majority of severely ill patients in the compassionate use program with infections caused by MDR and CR P. aeruginosa responded to cefiderocol treatment, even in cases where the cefiderocol MIC value was greater than the FDA susceptible breakpoint of 1 μg/mL. These findings provide clinical data that support the PK/PD modeling and animal efficacy data that were used to establish the CLSI susceptibility breakpoint of ≤4 μg/mL for P. aeruginosa. Overall, cefiderocol demonstrated good activity against isolates with either acquired carbapenemases or PDC variants that led to resistance to newer BL-BLI antibiotics. Despite the complicated nature of the patients in the compassionate use program, cefiderocol was generally well tolerated. Use of the CLSI cefiderocol susceptibility breakpoint, as opposed to the FDA susceptibility breakpoint, may provide additional patients with an effective and well-tolerated antibiotic treatment option for infections due to MDR and CR P. aeruginosa.

## MATERIALS AND METHODS

### Compassionate use program for cefiderocol.

The use of cefiderocol was permitted by the manufacturer after receiving requests from treating physicians, who obtained local approval from ethics committees or institutional review boards, in U.S., Canadian, and European hospitals. Emergency investigational new drug applications were requested from the FDA for U.S. cases. Informed consent was obtained from patients or their appropriate representatives at the time of the application. Individual patients were eligible for compassionate use of cefiderocol if they had a life-threatening infection due to a Gram-negative organism(s) for which no suitable alternative treatment option was available due to lack of activity, contraindications, allergies, or established toxicity of available antibiotics.

As part of the application for compassionate use, the treating physicians provided the patient’s age, sex, body weight, renal function, comorbidities, and relevant medical history. Infection diagnosis was described along with the date of onset. The causative pathogen with its local antimicrobial susceptibility profile, contraindications to potential antimicrobial therapies, and concomitant medications were also provided by the treating physicians.

### Cefiderocol dosing.

Cefiderocol 2 g was administered every 8 h in 3-h infusions for patients with normal renal function or with mild renal impairment. For patients with moderate or severe renal function impairment or augmented renal clearance, cefiderocol dosage was adjusted according to renal function, as proposed in the now-approved product label (13, 14).

### P. aeruginosa isolates.

Isolates of P. aeruginosa and other Gram-negative pathogens from patients in the compassionate use program were sent, if they were available, to the central laboratory (International Health Management Associates [IHMA], Schaumburg, IL, USA) for species confirmation with matrix-assisted laser desorption ionization–time of flight mass spectrometry (Bruker Daltonics, Billerica, MA, USA) and antimicrobial susceptibility testing to cefiderocol and nine other agents (amikacin, aztreonam, cefepime, ceftazidime-avibactam, ceftolozane-tazobactam, ciprofloxacin, colistin, imipenem, and meropenem). Concurrent Gram-positive bacteria, fungal species, and other pathogens (e.g., Mycobacterium spp.) were identified and tested only locally.

Antimicrobial susceptibility testing was determined by the reference broth microdilution method using Chelex-treated, iron-depleted, cation-adjusted Mueller-Hinton broth for cefiderocol and cation-adjusted Mueller-Hinton broth (BBL; Becton, Dickinson, Sparks, MD, USA) for other agents according to CLSI guidelines on single panels (21, 37). Details of chelation, determination of iron concentration, quality control testing, and preparation of broth microdilution panels have been described previously (38).

For this analysis, antimicrobial susceptibility was interpreted according to CLSI and FDA criteria for cefiderocol and CLSI criteria for other antibiotics, which are recognized by the FDA, with the exception of cefepime and colistin (20, 21). If the isolate was not susceptible to either meropenem or imipenem, it was categorized as CR (21). MDR P. aeruginosa isolates were those that were not susceptible to ≥1 antimicrobial agent in ≥3 antibiotic categories (i.e., polymyxin [colistin], antipseudomonal carbapenem [meropenem, imipenem], cefepime, monobactam [aztreonam], antipseudomonal fluoroquinolone [ciprofloxacin], or aminoglycoside [amikacin]) (39).

### Molecular characterization.

P. aeruginosa isolates underwent molecular characterization for β-lactamase genes by PCR at the central laboratory (IHMA) as previously described (16, 17). In brief, genomic DNA from all P. aeruginosa isolates was extracted using the QIAamp DNA Mini protocol for the QIAcube (Qiagen, Gaithersburg, MD, USA) according to the instructions provided by the manufacturer. P. aeruginosa isolates were screened for the presence of β-lactamase genes encoding ESBLs (*bla*_TEM_; *bla*_SHV_; *bla*_CTX-M_ enzymes, including five subtypes [*bla*_CTX-M-1-type_, *bla*_CTX-M-2-type_, *bla*_CTX-M-8-type_, *bla*_CTX-M-9-type_, and *bla*_CTX-M-25-type_]; *bla*_GES_ [all variants]; *bla*_VEB_; and *bla*_PER_ [including *bla*_PER-1-like_ and *bla*_PER-2-like_ subtypes]), carbapenemases (*bla*_KPC_, *bla*_OXA-24/40-like_ group, *bla*_NDM_, *bla*_IMP_, *bla*_VIM_, *bla*_SPM_, and *bla*_GIM_), and acquired *bla*_AmpC_ β-lactamases (*bla*_ACC_, *bla*_ACT_, *bla*_CMY_, *bla*_DHA_, *bla*_FOX_, *bla*_MIR_, and *bla*_MOX_) using published primers (40, 41). The intrinsic chromosomal *bla*_AmpC_ PDC (*bla*_PDC_) in P. aeruginosa was amplified with unpublished custom primers PDC-US-40f (5′-CGGTTTCTCATGCAGCCAAC-3′) and PDC-DS+1234r (5′-ACCATCATAGCCAGGACCG-3′). All genes detected in multiplex reactions were reamplified in full using extragenic primers, and both DNA strands were sequenced. The deduced amino acid sequence was compared with available databases maintained by the National Center for Biotechnology Information (NCBI; https://www.ncbi.nlm.nih.gov/) to identify curated enzyme variants.

During identification of PDC variants against NCBI databases, any new combination of mutations without a variant number was labeled as a “new variant.” Identified mutations and the respective amino acid variations were grouped based on previously published groupings (see Table S5 in the supplemental material) (34). Sequence numbering includes the signal peptide.

### Outcomes.

For analysis of clinical response rates by MIC value, baseline P. aeruginosa isolates were selected prior to initiation of cefiderocol treatment. If multiple baseline P. aeruginosa isolates were present, the organism with the highest cefiderocol MIC value was selected.

Following initiation of treatment, clinical response, vital status, and adverse events were reported by the treating physicians. Clinical response (based on signs and symptoms relevant to each infection type) was evaluated by the treating physician upon monitoring the patient over the course of treatment. The definition of clinical response and the minimum or the maximum duration of treatment were not prespecified by the manufacturer. An overall clinical response was reported as “response,” “improvement,” “stabilization,” or “cure and discharge.” If there was no clinical response, “no response” was reported. If during follow-up request, no further information was provided in case of missing response information, clinical response was marked as “unknown.” The source of the P. aeruginosa isolates was defined as respiratory tract infection, bloodstream infection, urinary tract infection, skin and soft tissue infection, osteomyelitis, prosthetic joint infection, spondylodiscitis, and gastrointestinal tract infection. Serious adverse event reporting was mandatory. Vital status information was spontaneously reported or requested through a follow-up from the treating physician. The impact of cefiderocol MIC based on both CLSI and FDA interpretive criteria on clinical response was evaluated. Only descriptive statistics were collected, including the number and percentage of patients for categorical parameters and median and range (minimum and maximum) for continuous parameters. For the clinical response rates, within-group 95% confidence intervals were calculated by the Clopper-Pearson method.

### Data availability.

All analyzed clinical, *in vitro* activity, and molecular data are included in the manuscript or the supplemental material. Other types of data may be available from the corresponding author at reasonable request made by researchers and investigators. A further data-sharing policy by Shionogi related to clinical studies can be found at Shionogi’s portal site (https://www.shionogi.com/global/en/company/policies/clinical-trial-data-transparency-policy.html).
