# Supplementary material for: Cefiderocol Treatment for Patients with Multidrug- and Carbapenem-Resistant Pseudomonas aeruginosa Infections in the Compassionate Use Program
Source: Antimicrob Agents Chemother. 2023 Jun 22;67(7):e00194-23. doi: 10.1128/aac.00194-23 (PMC10353454; doi:10.1128/aac.00194-23)
Supplement: Supplemental file 1 — Supplemental material. Download aac.00194-23-s0001.docx, DOCX file, 0.2 MB [file aac.00194-23-s0001.docx]

**FIG S1** Cefiderocol MIC distribution in 46 multidrug- or carbapenem-resistant *Pseudomonas aeruginosa* isolates based on ceftolozane-tazobactam susceptibility status.


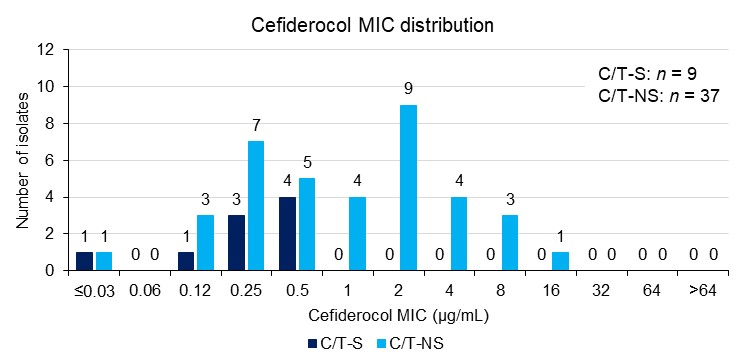


C/T, ceftolozane-tazobactam; NS, non-susceptible (i.e., intermediate, resistant); S, susceptible.

**FIG S2** Cefiderocol MIC distribution in 46 multidrug- or carbapenem-resistant *Pseudomonas aeruginosa* isolates based on ceftazidime-avibactam susceptibility status.


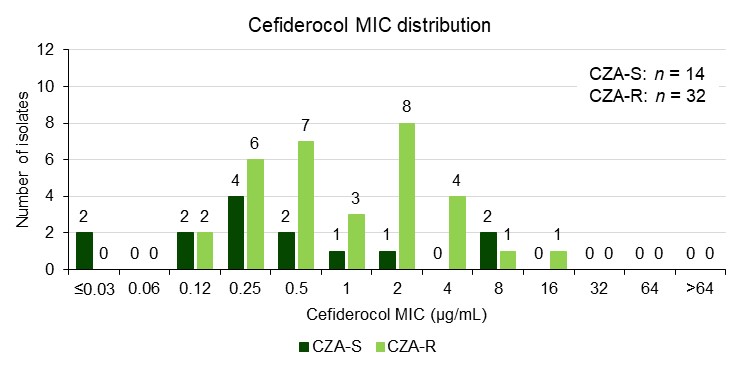


CZA, ceftazidime-avibactam; R, resistant; S, susceptible.

**TABLE S1** Cefiderocol susceptibility testing interpretive criteria of the US Food and Drug Administration, Clinical and Laboratory Standards Institute, and European Committee on Antimicrobial Susceptibility Testing

|  | **MIC (µg/mL)** | | |
| --- | --- | --- | --- |
|  | **Susceptible** | **Intermediate** | **Resistant** |
| US FDA | ≤1 | 2 | ≥4 |
| CLSI | ≤4 | 8 | ≥16 |
| EUCAST | ≤2 | - | >2 |

CLSI, Clinical and Laboratory Standards Institute; EUCAST, European Committee on Antimicrobial Susceptibility Testing; FDA, US Food and Drug Administration.

**TABLE S2** Antimicrobial susceptibility profiles of 46 *Pseudomonas aeruginosa* isolates from patients under compassionate use program with molecular characterization and concomitant and prior antibiotics

| **Patient #** | **Infection site of sample** | **FDC MIC (µg/mL)** | **C/T MIC (µg/mL)** | **CZA MIC (µg/mL)** | **MEM MIC (µg/mL)** | **IMI MIC (µg/mL)** | **AMK MIC (µg/mL** | **AZT MIC (µg/mL)** | **CEF MIC (µg/mL)** | **CFX MIC (µg/mL)** | **COL MIC (µg/mL)** | | **Acquired β-lactamases** | | **PDC variant** | | **Amino acid substitutions in PDC^a^** | | **Concomitant antibacterial agents** | | **Prior antibacterial agents (within ~90 days)** | |
| --- | --- | --- | --- | --- | --- | --- | --- | --- | --- | --- | --- | --- | --- | --- | --- | --- | --- | --- | --- | --- | --- | --- |
| Cefiderocol susceptibility: susceptible by FDA and CLSI | | | | | | | | | | | |  | |  | |  | |  | |  | |  |
| 22 | Lung | **0.03** | 8 | 1 | 8 | 32 | 64 | 16 | >16 | >4 | 1 | |  | | PDC-225 | | T105A, G27D | | Not clearly defined: maybe linezolid | | Ceftolozane-tazobactam, meropenem, polymyxin B | |
| 37 | Lung | **0.03** | 0.5 | 2 | 2 | 8 | 4 | 2 | 8 | 4 | 2 | |  | | PDC-19A | | T105A, G27D, V205L, G391A, V356I | | Meropenem, minocycline, | | Meropenem, minocycline, | |
| 6 | Lung | **0.12** | 64 | 16 | 64 | 32 | >64 | 16 | >16 | >4 | 8 | |  | | NA | | - | | Azithromycin, cefepime, inhaled colistin, eravacycline | | Azithromycin, cefepime, eravacycline, inhaled colistin, inhaled tobramycin | |
| 10 | GI | **0.12** | 16 | 64 | 64 | 32 | 8 | >32 | >16 | >4 | 1 | |  | | PDC-19A | | T105A, G27D, V205L, G391A, V356I | | Linezolid, metronidazole | | Amikacin, ceftazidime-avibactam, levofloxacin, piperacillin-tazobactam polymyxin B | |
| 27 | Urine | **0.12** | 64 | 4 | 4 | 16 | >64 | 16 | >16 | >4 | 0.5 | | GES-1 | | PDC-35 | | T105A, G27D, A98V, V205L, G391A | | Not clearly defined; maybe daptomycin, meropenem | | Azithromycin, aztreonam, ceftriaxone, colistin, daptomycin, meropenem, piperacillin-tazobactam, polymyxin B, vancomycin | |
| 29 | Lung | **0.12** | 1 | 4 | 0.25 | 1 | 16 | 8 | 8 | 0.25 | 1 | |  | | PDC-5 | | T105A, R79Q | | Eravacycline | | Amikacin, azithromycin, clarithromycin, colistin, delafloxacin, eravacycline, imipenem, meropenem, meropenem-vaborbactam, tedizolid, trimethoprim-sulfamethoxazole | |
| 4 | Wound | **0.25** | >64 | >64 | >64 | >64 | 64 | 16 | 16 | >4 | 2 | | VIM-2 | | PDC-3 | | T105A | | Cefepime, colistin, metronidazole | | Amikacin, polymyxin B | |
| 5 | GI | **0.25** | >64 | 64 | 4 | 2 | 4 | >32 | >16 | >4 | 1 | |  | | PDC-446 | | T105A, R79Q, E247K | | Linezolid, meropenem, metronidazole, polymyxin B, rifampicin | | Meropenem, polymyxin B, rifampicin | |
| 9 | Wound (BJI) | **0.25** | 0.5 | 1 | 0.5 | 2 | 8 | 4 | 2 | >4 | 2 | |  | | PDC-19A | | T105A, G27D, V205L, G391A, V356I | | Cefepime, daptomycin, eravacycline, polymyxin B | | Cefepime, ceftaroline, daptomycin, eravacycline, polymyxin B | |
| 15 | GI | **0.25** | 16 | 8 | >64 | >64 | 16 | 16 | 8 | >4 | 2 | |  | | PDC-5 | | T105A, R79Q | | Ceftazidime-avibactam, daptomycin, piperacillin-tazobactam | | Ceftazidime-avibactam, ceftolozane-tazobactam, colistin, meropenem, metronidazole, piperacillin-tazobactam, vancomycin | |
| 32 | Lung | **0.25** | 32 | 8 | >64 | >64 | 8 | >32 | >16 | >4 | 2 | | NDM-1 | | PDC-103 | | T105A, P82L | | Not clearly defined, maybe amikacin, ceftazidime-avibactam, polymyxin B | | Ceftazidime-avibactam, ceftolozane-tazobactam, inhaled + IV amikacin, meropenem, polymyxin B, vancomycin | |
| 35 | Lung | **0.25** | >64 | 64 | 8 | 2 | 32 | 16 | 16 | 1 | 0.5 | |  | | PDC-New Variant-9 | | T105A, L176R, I182F, G183D, K232E, V356I | | Not clearly defined; maybe colistin | | Ceftolozane-tazobactam, ciprofloxacin, IV and inhaled colistin, linezolid, meropenem, tobramycin | |
| 38 | Lung | **0.25** | 2 | 16 | 16 | 16 | 4 | 32 | 16 | 2 | 2 | |  | | PDC-3 | | T105A | | Inhaled colistin, tobramycin | | Ceftolozane-tazobactam, inhaled colistin, piperacillin-tazobactam, meropenem, tobramycin | |
| 40 | Wound | **0.25** | >64 | >64 | >64 | >64 | >64 | 16 | >16 | >4 | 1 | | IMP-15, VIM-2 | | PDC-3 | | T105A | | Piperacillin-tazobactam, polymyxin B | | Colistin, polymyxin B | |
| 42 | Wound (OM) | **0.25** | 4 | 8 | 16 | 32 | 16 | >32 | 16 | >4 | 1 | |  | | PDC-16 | | T105A, G27D, V205L, G391A | | Daptomycin, meropenem, metronidazole, tobramycin; later daptomycin, ertapenem | | Meropenem, tobramycin | |
| 45 | Blood | **0.25** | >64 | >64 | >64 | >64 | 64 | 32 | >16 | >4 | 1 | | IMP-18, VIM-2, GES-1 | | PDC-3 | | T105A | | Not reported | | Not reported | |
| 2 | Wound (OM) | **0.5** | 1 | 4 | 16 | UNK | UNK | UNK | >64 | >8 | 2 | |  | | PDC-New Variant-2 | | T105A, R324del | | Trimethoprim-sulfamethoxazole, vancomycin | | Aztreonam, colistin | |
| 3 | Wound | **0.5** | 4 | 32 | 32 | UNK | UNK | UNK | 32 | 8 | 1 | |  | | PDC-5 | | T105A, R79Q | | Levofloxacin, inhaled tobramycin, meropenem, metronidazole, minocycline, oral vancomycin, tedizolid, trimethoprim- sulfamethoxazole | | Piperacillin-tazobactam | |
| 11 | Blood | **0.5** | 8 | 64 | 8 | 8 | 4 | >32 | >16 | >4 | 2 | |  | | PDC-3 | | T105A | | Meropenem, tobramycin, vancomycin | | Gentamicin, meropenem, tobramycin | |
| 13 | Blood | **0.5** | >64 | 32 | 8 | 32 | 32 | >32 | >16 | >4 | 2 | | PER-1 | | PDC-8 | | T105A, L176R | | IV Bacteriophages, vancomycin | | Colistin, meropenem | |
| 20 | Lung | **0.5** | 4 | 1 | 16 | 32 | 8 | >32 | >16 | >4 | 0.5 | |  | | PDC-New Variant-7 | | R79Q, Q117W | | Bedaquiline, colistin, inhaled amikacin, inhaled aztreonam, linezolid | | Colistin | |
| 24 | Urine | **0.5** | >64 | >64 | >64 | >64 | 16 | >32 | >16 | >4 | 0.5 | | GES-40 | | PDC-19A | | T105A, G27D, V205L, G391A, V356I | | None | | Colistin, gentamicin, levofloxacin, meropenem, trimethoprim | |
| 30 | GI | **0.5** | >64 | 32 | 64 | >64 | 32 | 16 | 16 | >4 | 1 | | VIM-2 | | PDC-3 | | T105A | | Not clearly defined: maybe aztreonam | | Aztreonam, colistin, metronidazole, piperacillin-tazobactam | |
| 41 | Lung | **0.5** | 4 | 32 | 32 | 16 | 4 | >32 | >16 | >4 | 1 | |  | | PDC-120 | | T105A, G391A | | Ceftolozane-tazobactam, polymyxin B | | IV + inhaled tobramycin, meropenem, ceftolozane-tazobactam, colistin, polymyxin B, vancomycin | |
| 44 | Urine | **0.5** | >64 | 32 | 64 | >64 | >64 | 16 | 16 | >4 | 2 | | VIM-2 | | PDC-3 | | T105A | | Colistin, piperacillin-tazobactam | | Ceftazidime, colistin, fosfomycin | |
| 7 | Wound | **1** | 16 | 16 | 16 | 16 | 4 | >32 | >16 | >4 | 1 | |  | | PDC-New Variant-3 | | T105A, G27D, V205L, G391A, M318L | | Amikacin, linezolid | | Amikacin, linezolid | |
| 8 | Lung | **1** | 64 | 8 | 16 | 32 | 64 | >32 | >16 | >4 | 1 | |  | | PDC-New Variant-4 | | T105A, R79Q, G242S | | Azithromycin, inhaled colistin, piperacillin-tazobactam | | Azithromycin, cefepime, inhaled colistin, meropenem, piperacillin-tazobactam | |
| 12 | Wound (BJI) | **1** | 32 | 16 | 16 | 8 | 4 | >32 | 16 | >4 | 1 | |  | | PDC-New Variant-5 | | T105A, G27D, V205L, G391A, F147L | | Daptomycin, meropenem, polymyxin B | | Ceftolozane-tazobactam, daptomycin, meropenem, polymyxin B | |
| 36 | Blood | **1** | >64 | 32 | 64 | 32 | 32 | >32 | >16 | >4 | 2 | | VEB-9 | | PDC-11 | | T105A, P7S, V205L, G391A | | Not reported | | Amikacin, cefepime, polymyxin B | |
| Cefiderocol susceptibility: non-susceptible by FDA and susceptible by CLSI | | | | | | | | | | | |  | |  | |  | |  | |  | |  |
| 16 | Lung | **2** | >64 | >64 | >64 | 32 | 4 | >32 | >16 | >4 | 1 | |  | | PDC-New Variant-6 | | T105A, L176R, E198K, V239A | | Ceftazidime-avibactam, daptomycin, polymyxin B | | Amikacin, aztreonam, cefepime, ceftazidime, ceftazidime-avibactam, colistin, daptomycin, linezolid, moxifloxacin, polymyxin B, tobramycin, vancomycin | |
| 17 | Lung | **2** | >64 | >64 | 16 | 8 | >64 | >32 | >16 | >4 | 0.5 | |  | | PDC-New Variant-6 | | T105A, L176R, E198K, V239A | | Ceftolozane-tazobactam, colistin, vancomycin | | Ceftolozane-tazobactam, inhaled and IV colistin piperacillin-tazobactam | |
| 19 | Blood | **2** | >64 | >64 | >64 | 32 | 8 | >32 | >16 | >4 | 1 | |  | | PDC-3 | | T105A | | Polymyxin B, vancomycin, | | Aztreonam, ceftazidime-avibactam, polymyxin B | |
| 21 | Urine | **2** | >64 | >64 | >64 | >64 | >64 | 16 | >16 | >4 | 1 | | NDM-1 | | PDC-19A | | T105A, G27D, V205L, G391A, V356I | | Aztreonam, ceftazidime-avibactam | | Aztreonam, ceftazidime-avibactam, colistin, meropenem | |
| 31 | Wound | **2** | >64 | >64 | 64 | 8 | 8 | >32 | >16 | >4 | 1 | |  | | PDC-35 | | T105A, G27D, A98V, V205L, G391A | | Not clearly defined; maybe amikacin, colistin | | Amikacin, ceftolozane-tazobactam, colistin, linezolid, minocycline | |
| 33 | Lung | **2** | 64 | 2 | 4 | 16 | >64 | 16 | 16 | 1 | 1 | | GES-7 | | PDC-8 | | T105A, L176R | | Not reported | | Colistin | |
| 34 | Blood | **2** | >64 | >64 | >64 | >64 | >64 | >32 | >16 | >4 | 2 | | NDM-1, VEB-9 | | PDC-11 | | T105A, P7S, V205L | | Maybe colistin, and meropenem | | Ceftazidime-avibactam, ceftolozane-tazobactam, colistin, clindamycin, daptomycin, meropenem | |
| 39 | Blood | **2** | >64 | 32 | 32 | 32 | 32 | >32 | >16 | >4 | 2 | | VEB-9 | | PDC-11 | | T105A, P7S, V205L, G391A | | Ceftazidime-avibactam, polymyxin B | | Cefepime, colistin, fosfomycin, meropenem, polymyxin B, vancomycin | |
| 43 | Wound | **2** | 64 | >64 | >64 | 8 | 16 | >32 | >16 | 4 | 1 | |  | | PDC-409 | | T105A, L176R, G183D | | Ceftolozane-tazobactam, erythromycin, metronidazole, tobramycin, vancomycin | | Cefazolin, ceftolozane-tazobactam, metronidazole, tobramycin, vancomycin | |
| 14 | Wound | **4** | >64 | >64 | >64 | >64 | >64 | 8 | >16 | >4 | 1 | | NDM-1 | | PDC-16 | | T105A, G27D, V205L, G391A | | Aztreonam, polymyxin B, tigecycline | | Aztreonam, cefepime, ceftazidime-avibactam, metronidazole, polymyxin B, tigecycline, vancomycin | |
| 18 | Lung | **4** | >64 | >64 | 16 | 2 | 8 | >32 | >16 | 2 | 0.5 | |  | | PDC-5 | | T105A, R79Q | | Amikacin, cefazolin | | Amikacin, colistin, doxycycline | |
| 25 | Lung | **4** | 64 | >64 | 16 | 2 | >64 | 32 | 16 | >4 | 2 | |  | | PDC-373 | | T105A, P7S, T96I, V205L, G391A, E247K | | Not clearly defined: maybe colistin, imipenem | | Colistin, imipenem | |
| 26 | Lung | **4** | >64 | >64 | 32 | 4 | 8 | >32 | >16 | 0.5 | 1 | |  | | PDC-335 | | F147L | | Not clearly defined: maybe levofloxacin, tobramycin | | Ceftolozane-tazobactam, colistin, levofloxacin, meropenem, tobramycin | |
| Cefiderocol susceptibility: not susceptible by FDA or CLSI | | | | | | | | | | | |  | |  | |  | |  | |  | |  |
| 23 | Blood | **8** | 64 | 16 | 16 | 16 | 4 | 32 | 16 | 0.5 | 1 | |  | | PDC-191 | | T105A, F147L | | Aztreonam, ceftazidime-avibactam, daptomycin, polymyxin B | | Aztreonam, ceftazidime-avibactam, ceftolozane-tazobactam, daptomycin, oral vancomycin, polymyxin B, trimethoprim-sulfamethoxazole | |
| 28 | Wound | **8** | 32 | 8 | 1 | 1 | 32 | 8 | 16 | >4 | 1 | | GES-1 | | PDC-35 | | T105A, G27D, A98V, V205L, G391A | | Not clearly defined: maybe meropenem, trimethoprim- sulfamethoxazole | | Aztreonam, ceftazidime, ceftazidime-avibactam, clindamycin, meropenem, oxacillin, trimethoprim-sulfamethoxazole, vancomycin | |
| 46 | Lung | **8** | 32 | 8 | 32 | 32 | 32 | 16 | >16 | 2 | 0.5 | |  | | PDC-97 | | T105A, V239A | | Not clearly defined | | Not clearly defined | |
| 1 | Lung | **16** | >64 | >64 | 64 | UNK | UNK | UNK | >64 | >8 | 0.5 | |  | | PDC-New Variant-1 | | T105A, R79Q, P180L, E247K | | Ampicillin/sulbactam | | Ampicillin-sulbactam, aztreonam, cefazolin, meropenem, minocycline, polymyxin B | |

^a^Sequence numbering encompasses signal peptide.
AMK, amikacin; AZT, aztreonam; BJI, bone and joint infection; CEF, cefepime; CFX, ciprofloxacin; CLSI, Clinical and Laboratory Standards Institute; COL, colistin; C/T, ceftolozane-tazobactam; CZA, ceftazidime-avibactam; FDA, Food and Drug Administration; FDC, cefiderocol; GES, Guiana extended-spectrum β-lactamase; GI, gastrointestinal; IMI, imipenem; IMP, imipenemase metallo-β-lactamase; IV, intravenous; MEM, meropenem; NA, not available; NDM, New Delhi metallo-β-lactamase; OM, osteomyelitis; PDC, *Pseudomonas*-derived cephalosporinase; PER, *Pseudomonas* extended resistance; UNK, unknown; VEB, Vietnamese extended-spectrum β-lactamase; VIM, Verona integron-encoded metallo-β-lactamase.

**TABLE S3** Susceptibility to β-lactam agents, and clinical presentation and outcomes for *Pseudomonas aeruginosa* isolates with *Pseudomonas*-derived cephalosporinase variants with Group 1–3 mutations

| **Patient #** | **PDC variant** | **MIC (µg/mL)** | | | | | | | **‘Berrazeg grouping’ amino acid variations (mutations) in PDC** | | | | | | | | | **Clinical presentation and outcomes** | | |
| --- | --- | --- | --- | --- | --- | --- | --- | --- | --- | --- | --- | --- | --- | --- | --- | --- | --- | --- | --- | --- |
|  |  | **FDC** | **C/T** | **CZA** | **MEM** | **IMI** | **AZT** | **CEF** | **Group 1** | **Group 2** | **Group 3** | **Other** | **Polymorphisms** | | | | | **Infection** | **Clinical response** | **Vital status** |
| **1** | **PDC-New Variant-1** | 16 | >64 | >64 | 64 | UNK | UNK | >64 | E247K |  | P180L |  | T105A | R79Q |  |  |  | RTI | Responded | Survived |
| **5** | **PDC-446** | 0.25 | >64 | 64 | 4 | 2 | >32 | >16 | E247K |  |  |  | T105A | R79Q |  |  |  | GI | Responded | Survived |
| **25** | **PDC-373** | 4 | 64 | >64 | 16 | 2 | 32 | 16 | E247K |  |  |  | T105A | P7S | T96I | V205L | G391A | RTI | Resolved | Survived |
| **8** | **PDC-New Variant-4** | 1 | 64 | 8 | 16 | 32 | >32 | >16 | G242S |  |  |  | T105A | R79Q |  |  |  | RTI | Responded | Died |
| **16** | **PDC-New Variant-6** | 2 | >64 | >64 | >64 | 32 | >32 | >16 | V239A |  |  |  | T105A | L176R | E198K |  |  | RTI | No response | Died |
| **17** | **PDC-New Variant-6** | 2 | >64 | >64 | 16 | 8 | >32 | >16 | V239A |  |  |  | T105A | L176R | E198K |  |  | RTI | Responded | Survived |
| **46** | **PDC-97** | 8 | 32 | 8 | 32 | 32 | 16 | >16 | V239A |  |  |  | T105A |  |  |  |  | RTI | Resolved | Survived |
| **7** | **PDC-New Variant-3** | 1 | 16 | 16 | 16 | 16 | >32 | >16 |  | M318L |  |  | T105A | G27D | V205L | G391A |  | Wound | Responded | Survived |
| **12** | **PDC-New Variant-5** | 1 | 32 | 16 | 16 | 8 | >32 | 16 |  |  | F147L |  | T105A | G27D | V205L | G391A |  | Wound | Unknown | Survived |
| **23** | **PDC-191** | 8 | 64 | 16 | 16 | 16 | 32 | 16 |  |  | F147L |  | T105A |  |  |  |  | Blood | Responded | Survived |
| **26** | **PDC-335** | 4 | >64 | >64 | 32 | 4 | >32 | >16 |  |  | F147L |  |  |  |  |  |  | RTI | Resolved | Survived |
| **20** | **PDC-New Variant-7** | 0.5 | 4 | 1 | 16 | 32 | >32 | >16 |  |  |  |  |  | R79Q | Q117**W** |  |  | RTI | Responded | Survived |
| **2** | **PDC-New Variant-2** | 0.5 | 1 | 4 | 16 | UNK | UNK | >64 |  |  |  | R324del | T105A |  |  |  |  | Wound, bone | Responded | Survived |

^a^Sequence numbering encompasses signal peptide.
AZT, aztreonam; CEF, cefepime; C/T, ceftolozane-tazobactam; CZA, ceftazidime-avibactam; FDC, cefiderocol; GI, gastrointestinal; IMI, imipenem; MEM, meropenem; PDC, *Pseudomonas*-derived cephalosporinase; RTI, respiratory tract infection; UNK, unknown.
Blue: Group 1 mutations; Purple: Group 2 mutations; Orange: Group 3 mutations; Cyan: substitution.

**TABLE S4** Distribution of cefiderocol, ceftolozane-tazobactam, and ceftazidime-avibactam MIC values stratified by PDC allele among multidrug- or carbapenem-resistant *P. aeruginosa* isolates without a carbapenemase, extended-spectrum β-lactamases, or acquired AmpC β-lactamase

| **MIC (µg/mL)** | **Antibiotic** | **PDC variant** | | | | | | | | | | | | | | | | | | | | | **Total** |
| --- | --- | --- | --- | --- | --- | --- | --- | --- | --- | --- | --- | --- | --- | --- | --- | --- | --- | --- | --- | --- | --- | --- | --- |
|  |  | **PDC-120** | **PDC-16** | **PDC-191** | **PDC-19A** | **PDC-225** | **PDC-3** | **PDC-35** | **PDC-373** | **PDC-335** | **PDC-409** | **PDC-446** | **PDC-5** | **PDC-97** | **PDC-New Variant-1** | **PDC-New Variant-2** | **PDC-New Variant-3** | **PDC-New Variant-4** | **PDC-New Variant-5** | **PDC-New Variant-6** | **PDC-New Variant-7** | **PDC-New Variant-9** |  |
| **≤0.03** | **FDC** |  |  |  | 1 | 1 |  |  |  |  |  |  |  |  |  |  |  |  |  |  |  |  | **2** |
|  | **C/T** |  |  |  |  |  |  |  |  |  |  |  |  |  |  |  |  |  |  |  |  |  | **0** |
|  | **CZA** |  |  |  |  |  |  |  |  |  |  |  |  |  |  |  |  |  |  |  |  |  | **0** |
| **0.06** | **FDC** |  |  |  |  |  |  |  |  |  |  |  |  |  |  |  |  |  |  |  |  |  | **0** |
|  | **C/T** |  |  |  |  |  |  |  |  |  |  |  |  |  |  |  |  |  |  |  |  |  | **0** |
|  | **CZA** |  |  |  |  |  |  |  |  |  |  |  |  |  |  |  |  |  |  |  |  |  | **0** |
| **0.12** | **FDC** |  |  |  |  |  |  |  |  |  |  |  |  |  |  |  |  |  |  |  |  |  | **0** |
|  | **C/T** |  |  |  | 1 |  |  |  |  |  |  |  | 1 |  |  |  |  |  |  |  |  |  | **2** |
|  | **CZA** |  |  |  |  |  |  |  |  |  |  |  |  |  |  |  |  |  |  |  |  |  | **0** |
| **0.25** | **FDC** |  | 1 |  | 1 |  | 1 |  |  |  |  | 1 | 1 |  |  |  |  |  |  |  |  | 1 | **6** |
|  | **C/T** |  |  |  |  |  |  |  |  |  |  |  |  |  |  |  |  |  |  |  |  |  | **0** |
|  | **CZA** |  |  |  |  |  |  |  |  |  |  |  |  |  |  |  |  |  |  |  |  |  | **0** |
| **0.5** | **FDC** | 1 |  |  |  |  | 1 |  |  |  |  |  | 1 |  |  | 1 |  |  |  |  | 1 |  | **5** |
|  | **C/T** |  |  |  | 2 |  |  |  |  |  |  |  |  |  |  |  |  |  |  |  |  |  | **2** |
|  | **CZA** |  |  |  |  |  |  |  |  |  |  |  |  |  |  |  |  |  |  |  |  |  | **0** |
| **1** | **FDC** |  |  |  |  |  |  |  |  |  |  |  |  |  |  |  | 1 | 1 | 1 |  |  |  | **3** |
|  | **C/T** |  |  |  |  |  |  |  |  |  |  |  | 1 |  |  | 1 |  |  |  |  |  |  | **2** |
|  | **CZA** |  |  |  | 1 | 1 |  |  |  |  |  |  |  |  |  |  |  |  |  |  | 1 |  | **3** |
| **2** | **FDC** |  |  |  |  |  | 1 | 1 |  |  | 1 |  |  |  |  |  |  |  |  | 2 |  |  | **5** |
|  | **C/T** |  |  |  |  |  | 1 |  |  |  |  |  |  |  |  |  |  |  |  |  |  |  | **1** |
|  | **CZA** |  |  |  | 1 |  |  |  |  |  |  |  |  |  |  |  |  |  |  |  |  |  | **1** |
| **4** | **FDC** |  |  |  |  |  |  |  | 1 | 1 |  |  | 1 |  |  |  |  |  |  |  |  |  | **3** |
|  | **C/T** | 1 | 1 |  |  |  |  |  |  |  |  |  | 1 |  |  |  |  |  |  |  | 1 |  | **4** |
|  | **CZA** |  |  |  |  |  |  |  |  |  |  |  | 1 |  |  | 1 |  |  |  |  |  |  | **2** |
| **8** | **FDC** |  |  | 1 |  |  |  |  |  |  |  |  |  | 1 |  |  |  |  |  |  |  |  | **2** |
|  | **C/T** |  |  |  |  | 1 | 1 |  |  |  |  |  |  |  |  |  |  |  |  |  |  |  | **2** |
|  | **CZA** |  | 1 |  |  |  |  |  |  |  |  |  | 1 | 1 |  |  |  | 1 |  |  |  |  | **4** |
| **16** | **FDC** |  |  |  |  |  |  |  |  |  |  |  |  |  | 1 |  |  |  |  |  |  |  | **1** |
|  | **C/T** |  |  |  | 1 |  |  |  |  |  |  |  | 1 |  |  |  | 1 |  |  |  |  |  | **3** |
|  | **CZA** |  |  | 1 |  |  | 1 |  |  |  |  |  |  |  |  |  | 1 |  | 1 |  |  |  | **4** |
| **32** | **FDC** |  |  |  |  |  |  |  |  |  |  |  |  |  |  |  |  |  |  |  |  |  | **0** |
|  | **C/T** |  |  |  |  |  |  |  |  |  |  |  |  | 1 |  |  |  |  | 1 |  |  |  | **2** |
|  | **CZA** | 1 |  |  |  |  |  |  |  |  |  |  | 1 |  |  |  |  |  |  |  |  |  | **2** |
| **64** | **FDC** |  |  |  |  |  |  |  |  |  |  |  |  |  |  |  |  |  |  |  |  |  | **0** |
|  | **C/T** |  |  | 1 |  |  |  |  | 1 |  | 1 |  |  |  |  |  |  | 1 |  |  |  |  | **4** |
|  | **CZA** |  |  |  | 1 |  | 1 |  |  |  |  | 1 |  |  |  |  |  |  |  |  |  | 1 | **4** |
| **>64** | **FDC** |  |  |  |  |  |  |  |  |  |  |  |  |  |  |  |  |  |  |  |  |  | **0** |
|  | **C/T** |  |  |  |  |  | 1 | 1 |  | 1 |  | 1 | 1 |  | 1 |  |  |  |  | 2 |  | 1 | **9** |
|  | **CZA** |  |  |  |  |  | 1 | 1 | 1 | 1 | 1 |  | 1 |  | 1 |  |  |  |  | 2 |  |  | **9** |

C/T, ceftolozane-tazobactam; CZA, ceftazidime-avibactam; FDC, cefiderocol; PDC, *Pseudomonas*-derived cephalosporinase.
Green: cefiderocol; orange: ceftolozane-tazobactam; blue: ceftazidime-avibactam.

**TABLE S5** Location of mutations in *Pseudomonas*-derived cephalosporinase

| **Group #** | **Location of mutation in PDC gene** |
| --- | --- |
| Group 0 | Polymorphisms in multiple regions not considerably influencing beta-lactam susceptibility |
| Group 1 | C-terminal region of omega loop or alpha helix |
| Group 2 | R2 loop |
| Group 3 | Conserved YSN loop (at the extremity of H-5 and in loop alpha 3-alpha 4) |
| Group 4 | C3/C4 carboxylate recognition region contributing to substrate binding |

PDC, *Pseudomonas*-derived cephalosporinase.
Adapted from [Berrazeg M, et al. 2015. Antimicrob Agents Chemother 59:6248–6255].

**References.**

Berrazeg M, Jeannot K, Ntsogo Enguéné VY, Broutin I, Loeffert S, Fournier D, Plésiat P. 2015. Mutations in β-lactamase AmpC increase resistance of *Pseudomonas aeruginosa* isolates to antipseudomonal cephalosporins. Antimicrob Agents Chemother 59:6248–6255. doi: 10.1128/AAC.00825-15.
